# CPB1 of *Aedes aegypti* Interacts with DENV2 E Protein and Regulates Intracellular Viral Accumulation and Release from Midgut Cells

**DOI:** 10.3390/v6125028

**Published:** 2014-12-16

**Authors:** Hong-Wai Tham, Vinod R. M. T. Balasubramaniam, Bimo Ario Tejo, Hamdan Ahmad, Sharifah Syed Hassan

**Affiliations:** 1 Virus-Host Interaction Research Group, Infectious Disease Laboratory, Jeffrey Cheah School of Medicine and Health Sciences, Monash University Malaysia, Jalan Lagoon Selatan, Bandar Sunway, 47500 Subang Jaya, Selangor, Malaysia; E-Mails: hwtha1@student.monash.edu (H.-W.T.); vrmt1@student.monash.edu (V.R.M.T.B.); 2 Department of Biotechnology and Neuroscience, Faculty of Life Science, Surya University, 15810 Tangerang, Banten, Indonesia; E-Mail: bimo.tejo@surya.ac.id (B.A.T.); 3 School of Biological Sciences, Universiti Sains Malaysia, 11800 USM, Pulau Pinang, Malaysia; E-Mail: hamdana@usm.my

**Keywords:** dengue virus, *Aedes aegypti*, carboxypeptidase, yeast two-hybrid

## Abstract

*Aedes aegypti* is a principal vector responsible for the transmission of dengue viruses (DENV). To date, vector control remains the key option for dengue disease management. To develop new vector control strategies, a more comprehensive understanding of the biological interactions between DENV and *Ae. aegypti* is required. In this study, a cDNA library derived from the midgut of female adult *Ae. aegypti* was used in yeast two-hybrid (Y2H) screenings against DENV2 envelope (E) protein. Among the many interacting proteins identified, carboxypeptidase B1 (CPB1) was selected, and its biological interaction with E protein in *Ae. aegypti* primary midgut cells was further validated. Our double immunofluorescent assay showed that CPB1-E interaction occurred in the endoplasmic reticulum (ER) of the *Ae. aegypti* primary midgut cells. Overexpression of CPB1 in mosquito cells resulted in intracellular DENV2 genomic RNA or virus particle accumulation, with a lower amount of virus release. Therefore, we postulated that in *Ae. aegypti* midgut cells, CPB1 binds to the E protein deposited on the ER intraluminal membranes and inhibits DENV2 RNA encapsulation, thus inhibiting budding from the ER, and may interfere with immature virus transportation to the trans-Golgi network.

## 1. Introduction

The *Aedes aegypti* mosquito is more widely dispersed today, exposing a third of the world’s population to the risk of infections with one or more of the four dengue virus serotypes (DENV1-4). DENV is a positive-sense, single-stranded RNA virus with a total genome size of approximately 11 kb. It belongs to the *Flavivirus* genus of the *Flaviviridae* family. In its host, the DENV genome is translated into three structural proteins, C, prM and E, and seven non-structural proteins, NS1, NS2a, NS2b, NS3, NS4a, NS4b and NS5, from a single open reading frame. The multiple proteins are then cleaved into individual components via proteolytic cleavage by either viral or host proteases. DENV assembly occurs in the rough endoplasmic reticulum (rER) [1]. Then, the virus is channeled into the trans-Golgi network (TGN) for maturation to occur [2,3], followed by virus release from the infected host cell.

Recently, there has been an increased interest in understanding viral tropism and the molecular activities of DENV in *Ae. aegypti* and *Ae. albopictus* [4,5,6,7]. Following a blood meal from an infected human, DENVs multiply in the midgut of the mosquito for seven to 14 days. The viruses are disseminated from the midgut and proliferate in the salivary gland, where the virus is transmitted to another host when the infective mosquito takes another blood meal. The vital role of mosquitoes in the transmission cycle of DENV makes vector control an important strategy in disease management. The use of pesticides, however, has resulted in various issues, which hinder the on-going dengue management efforts [8,9,10,11,12,13,14]. Other strategies for mosquito control must therefore be considered. Some of these include directing research towards determining mechanisms of viral survival, virulence and tropism in the *Ae. aegypti* mosquito.

In nature, the mosquito vectorial capacity has been linked to the mosquito midgut epithelial barrier, midgut escape barrier and salivary escape barrier [15]. These differentiated tissues are only present in adult mosquitoes. In this study, female adult *Ae. aegypti* midgut tissues were collected for cDNA library construction and then used for yeast two-hybrid (Y2H) screening to identify interacting dengue virus proteins.

We constructed the cDNA library using a homologous recombination-mediated approach with the Gal4 activation domain-based vector, pGADT7. This library was screened against the DENV2 E protein, resulting in the identification of a number of interacting proteins, with carboxypeptidase B1 (CPB1) as one of the predominant DENV2 E protein interaction partners. To date, there have been no studies reporting the possible role of *Ae. aegypti* CPB1 protein during DENV infection. This study reports the molecular interactions between *Ae. aegypti* CPB1 and the DENV2 E protein, as well as the role of CPB1 in regulating viral replication and release from *Ae. aegypti* primary midgut cells.

## 2. Materials and Methods

### 2.1. Virus and Cell Cultures

Dengue virus type 2 (MY89-88549, AJ556804) was kindly provided by Prof. Sazaly Abu Bakar, University of Malaya. The virus was propagated in C6/36 (ATCC^®^ CRL-1660™) and Vero cells (ATCC^®^ CCL‑81™) until cytopathic effects were observed. The titer of DENV2 grown in C6/36 and Vero cells was determined in Vero cells and was 10^4^ and 10^6^ median tissue culture infective dose/mL (TCID_50_/mL), respectively. Vero cells were incubated at 37 °C with 5% CO_2_, in minimal essential medium (MEM) supplemented with 10% fetal bovine serum (FBS), 1% PSK (working concentration of 0.5 U/mL of penicillin “G” sodium, 0.5 mg/mL of streptomycin and 1 mg/mL kanamycin sulfate) and 1% HEPES buffer (Gibco^®^, Life Technologies, Carlsbad, CA, USA). The C6/36 cells were incubated at 28 °C with 5% CO_2_, maintained in minimal essential medium (MEM) identical to the supplements described above, with 1.5% HEPES buffer. Both cell lines were passaged once every 4–5 days.

### 2.2. Plasmid Construction and Yeast Transfection

The DENV2 envelope (E) gene (forward primer: AATGCGTTGTATAGGAATATC; reverse primer: AACCACTATCGGCCTGCACCAT) was cloned in-frame into the pGBKT7 plasmid (Clontech, Mountain View, CA, USA) and named pGBKT7-E. The plasmid was transfected into the yeast strain Y2HGold using the lithium/acetate method described previously [16]. Positively-transfected yeast colonies were maintained on selective agars with depleted tryptophan (SD/-Trp, synthetic dropout media without tryptophan) and cryopreserved at −80 °C until use.

### 2.3. Aedes aegypti Mosquitoes

*Ae. aegypti* mosquitoes (Linnaeus) [17,18] were maintained in the insectarium at the Universiti Sains Malaysia (USM), School of Biological Sciences, at 28 ± 1 °C under 70%–75% relative humidity, with a light/dark cycle of 14 h/10 h. The larvae were reared in round trays with mineral water (approximately 3 cm height) and provided with cat food (chicken sourced). The adults were fed with sterilized 10% sucrose solution *ad libitum*.

### 2.4. Aedes aegypti Primary Midgut Cell Preparations

Mosquitoes were cold anaesthetized, and all dissections were performed under sterile conditions following previously described protocols [19,20] with minor modifications. In brief, the mosquitoes were surface sterilized with a 70% ethanol solution [21] and rinsed with sterile phosphate-buffered saline (PBS). *Ae. aegypti* midguts were carefully dissected [22] under a dissecting microscope (15× magnification). The midguts were immersed in sterile PBS solution supplemented with 1× penicillin‑streptomycin-neomycin (PSN) antibiotic mixture (Gibco^®^, Life Technologies) and trypsinized with 1× Krebs-Ringer bicarbonate solution supplemented with 0.25% trypsin (37 °C for 25 minutes). The cells were pelleted (500× *g*, 4 °C, 15 min), resuspended in MEM media supplemented with 10% FBS and incubated in 96-well plates (28 °C, 5% CO_2_) with one passage every 12 days.

### 2.5. cDNA Library Construction

A total of 50 dissected *Ae. aegypti* primary midguts [22] were subjected to total RNA extraction using TRIzol® Reagent (Invitrogen™, Life Technologies). Following the manufacturer’s instructions and previously reported studies [23,24], first- and second-strand cDNA were synthesized using the Make Your Own “Mate & Plate™” Library System (Clontech). Double-stranded cDNA was purified, and nucleic acids less than 400 bp were discarded using Chroma Spin™ TE-400 Columns (Clontech). About 4.16 µg (10 µL) of purified double-stranded cDNA, in conjunction with 3 µg linearized pGADT7-rec vector and 200 µg denatured Yeastmaker Carrier DNA (Clontech) were mixed and transformed concurrently into competent yeast Y187 cells [16]. Then, the pelleted cells were re‑suspended in 15 mL sterile 0.9% (w/v) NaCl solution prior to spreading on selection agar plates with depleted leucine (SD/-Leu). To detect transformation efficiency, 100 µL was spread in dilutions of 10^−1^, 10^−2^ and 10^−3^ on 100-mm SD/-Leu agar plates, and others were spread on 150-mm plates for 150 µL each (a total of 100 plates). After incubation at 30 °C for 96 hours, the surviving colonies were counted to calculate the library titer and the number of independent clones [25]. Then, the plates were harvested with 5 mL YPDA (yeast extract solution with peptone, dextrose, and adenine) freezing medium each. All liquids were collected in a sterile 1-L flask and mixed well. After that, they were stored in 1-mL or 50-mL aliquots at −80 °C until use.

### 2.6. Yeast Two-Hybrid Screening

Y2H screening was conducted according to previous studies [26,27] and the manufacturer’s instructions. Prior to mating, Y2HGold was plated on SD/-Ade/-His/-Trp agar supplemented with X-α-gal (40 µg/mL) for the auto-activation test. In the absence of the pGADT7 plasmid, no auto-activation of any reporter was detected in our Y2HGold harboring pGBKT7-E. Yeast mating was conducted aseptically by inoculating an overnight 5-mL Y2Hgold culture (bait) with a 1-mL library aliquot (prey). The mixture was diluted to 50 mL using 2× YPDA broth. Mating was allowed for 24 hours at 30 °C with 45-rpm orbital shaking. Then, the yeast cells were pelleted and spread on low-stringency selection agar SD/-Leu/-Trp (DDO) in the presence of Aureobasidin A (Aba, 125 ng/mL) and X-α-gal, followed by high-stringency selection agar SD/-Leu/-Trp/-Ade/-His (QDO) supplemented with Aba and X-α-gal. After a 96-hour incubation at 30 °C, the plasmids were extracted from the surviving blue yeast colonies and sequenced for cDNA insert identification (BLAST).

### 2.7. Mammalian Two-Hybrid Analysis

**Figure 1:**
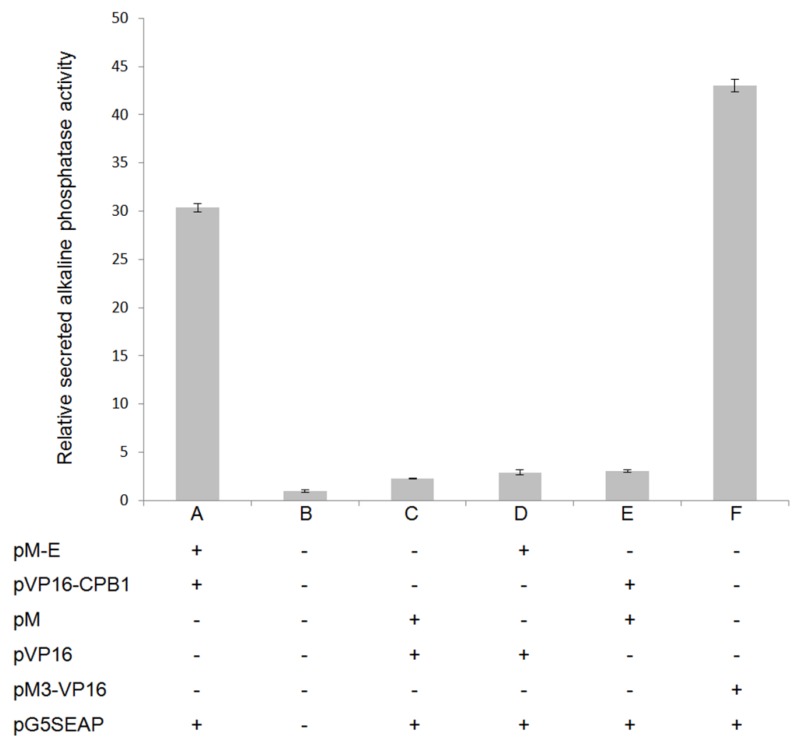
Mammalian two-hybrid assays confirm the physical interaction of CPB1 and E protein in Vero cells. The columns show the mean values and the error bars are the SD of three samples (*n* = 3). Each column represents a treatment and is labelled “**+**” for the plasmid transfected and “-” for the plasmid that is absent. (**A**) Interaction between CPB1 and E proteins. The secreted alkaline phosphatase (SEAP) activity is significantly higher than the negative controls. (**B**–**E**) Various negative controls in the presence/absence of different combinations of plasmid construct. (**F**) Positive controls transfected with pM3-VP16, which expresses a fusion of the GAL4 DNA-binding domain to the VP16 activation domain. Therefore, the positive controls had the highest SEAP activity. The media was harvested 48 hours post-transfection.

Mammalian two-hybrid analysis was performed as a complementary approach to the Y2H [28]. The cDNA of DENV2 E and *Ae. aegypti* CPB1 were cloned in-frame into the pM (GAL4 DNA-BD) and pVP16 (AD) cloning vectors (Matchmaker Mammalian Assay Kit 2, Clontech), respectively. Using the CalPhos™ Mammalian Transfection Kit (Clontech), Vero cells were co-transfected with different plasmid cocktails consisting of 5 µg each pM-E, pVP16-CPB1 and pG5SEAP according to the scheme in Figure 1. The transfected cells were incubated (37 °C, 5% CO_2_, 48 hours), and the culture media were harvested for SEAP (secreted alkaline phosphatase) activity analysis using the GreatEscAPe™ SEAP Chemiluminescence Detection Kit (Clontech). Chemiluminescent signals were detected using a PerkinElmer VICTOR™ X5 Multilabel Plate Reader. This assay was conducted in biological triplicates.

### 2.8. Double Immunofluorescence Assay

This assay was performed using a previously-described procedure [29] with minor modifications. Anti-E (GeneTex, GTX43296) and anti-CPB1 antibody (Abnova, H00001360-D01) were obtained commercially. *Ae. aegypti* primary midgut cells were infected with DENV2 at multiplicity of infection (MOI) of 10, and viral proliferation was allowed for 48 hours. Live cells were stained with ER‑Tracker™ Blue-White Dapoxyl™ (1 µM/mL) (Molecular Probes®, Life Technologies™), fixed (4% paraformaldehyde, 1× Hank's Balanced Salt Solution (HBSS), 10 min), permeabilized (0.25% Triton X-100, 1× HBSS, 10 min), blocked (1% BSA, 1× HBSS, 1 hour) and incubated with primary (anti-E and anti-CPB1 antibodies, 1:2000 dilution, 1 hour) and secondary antibodies (AlexaFluor^®^ 488, AlexaFluor^®^ 594, 1:2000 dilution, 1 hour). The slides were mounted and viewed under an IX81 confocal microscope (Olympus). This assay was conducted at room temperature, unless otherwise stated.

### 2.9. Co-Immunoprecipitation Analysis

*Ae. aegypti* CPB1 and DENV2 E protein were co-immunoprecipitated using the Dynabeads^®^ Co‑Immunoprecipitation Kit (Invitrogen™, Life Technologies) following the manufacturer’s instructions. In brief, DENV2-infected (MOI: 1) *Ae. aegypti* primary midgut cells were pelleted and lysed with lysis buffer (110 mM KOAc, 0.5% Triton X-100, 100 mM NaCl, 2 mM MgCl_2_, 1 mM DTT and 0.8 mM PMSF, pH 7.4). Anti-CPB1 and anti-E antibody-coupled resins were prepared according to the manufacturer’s instructions. In two independent experiments, antibody-coupled resins were mixed with cell lysates on a tube rotator (4 °C, 30 min). The resin-antibody-proteins complexes were collected using magnetic stands. The complexes were washed three times (0.05% Tween-20, 100 mM NaCl, 2 mM MgCl_2_ and 0.8 mM PMSF, pH 7.5) before the final elution of the protein complexes. The eluents were subjected to western blot assay with both primary antibodies, anti-E antibody (Abnova, MAB8902) and anti-CPB1 antibody (Abnova, H00001360-D01). Alkaline phosphatase (AP)-conjugated anti-rabbit antibody (host: goat) was used as the secondary antibody. The bands were visualized after incubation with the 5-bromo-4-chloro-3-indolyl-phosphate/nitro blue tetrazolium (BCIP/NBT) substrate. In parallel, 10% cellular lysate and non-antibody-coated beads were included in the western blot assays.

### 2.10. Molecular Docking Analysis

The three-dimensional structure of the DENV E was obtained from the Protein Data Bank (PDB: 1OAN). For *Ae. aegypti* CPB1, the protein structure was predicted using Iterative Threading ASSEmbly Refinement (I-TASSER) [30,31]. The PatchDock algorithm [32] was used to determine a rigid docking model between the CPB1 and E protein. The three-dimensional transformations of the Cartesian coordinate of the proteins were further refined using the FireDock algorithm [33,34]. The complex structure with the lowest interaction energy was used for further analyses.

### 2.11. CPB1 Upregulation Study

CPB1 upregulation studies were conducted in insect [35] and mammalian cell lines [36], C6/36 and Vero cells, respectively. *Ae. aegypti* CPB1 cDNA was cloned in-frame into the pIB/V5-His and pcDNA3.1(+) plasmids (Invitrogen™, Life Technologies). The cells were seeded on 24-well plates at 3 × 10^5^ cells/well one day before transfection. A total of 2 µg of plasmid constructs were transfected into each respective well using Lipofectamine^®^ 2000 transfection reagent (Invitrogen™, Life Technologies). CPB1 expression was allowed (28 °C, 5% CO_2_, 48 hours) before DENV2 infection (MOI: 1). After 48 hours, the cells and media were harvested for RNA extraction using the RNeasy Kit (Qiagen, Valencia, CA, USA). The RNA concentration and quality were determined using a NanoPhotometer® (Implen, München, Germany). The cDNA was constructed with the RevertAid Premium First-strand cDNA Synthesis Kit (Fermentas, Leicester, U.K.). Quantitative real-time PCR (qPCR) was conducted using a StepOnePlus qPCR System (Applied Biosystem®, Life Technologies) with the Power SYBR® Green PCR Master Mix (Applied Biosystems®, Life Technologies). The temperature cycle was as follows: 95 °C, 10 min; 40 cycles of 95 °C, 15 s, 60 °C, 30 s, and 72 °C, 40 s. A melt curve setting was included. The relative quantification of DENV2 genomic RNA was assessed using the comparative C_T_ (∆∆C_T_) algorithm [37]. This assay was conducted in triplicate. Results expressed as ΔΔC_T_ were reported as the mean standard deviation and analyzed using paired Student’s *t*-test. *p* values <0.05 were considered statistically significant.

## 3. Results 

### 3.1. Ae. aegypti Midgut cDNA Library Construction

The library titer and the total number of independent clones were calculated according to previous reported formulas [23,25]. Based on the colony number on SD/-Leu plates, the Y187 library titer (cfu/mL) was determined at 7.5 × 10^4^, which met the minimum requirement standard (6.7 × 10^4^ cfu/mL) of the manufacturer (Clontech). The total number of independent clone, which was an indication of the cDNA library complexity, was 3.75 × 10^7^. 

### 3.2. CPB1 Protein Interacts with the DENV2 E Protein

Using the Y2H assays, a total of 265 surviving blue yeast colonies were obtained from low‑stringency selection agars (SD/-Leu/-Trp). This number was reduced to 56 by eliminating false‑positive interactors on high-stringency selection agars (SD/-Leu/-Trp/-His/-Ade). The final step of the assay, including the sequencing of these colonies, resulted in 11 positive candidate proteins, because of multiple copies of sequences of frequently-interacting protein candidates. CPB1 was identified in seven of 56 yeast colonies surviving on high-stringency selecting agars. 

A mammalian two-hybrid (M2H) assay was also performed as a complementary experiment to the Y2H to confirm the interactions. Vero cells were co-transfected with the pM and pVP16 plasmids along with a pG5SEAP reporter vector, according to the scheme in Figure 1 (the full experimental procedure is described in the Materials and Methods section). pG5SEAP is transcriptionally activated by physical interaction between pM- and pVP16-conjugated proteins and expresses SEAP (secreted alkaline phosphatase), which can be determined maximally 48 hours following transfection. The SEAP reporter gene encodes alkaline phosphatase without the membrane-anchoring domain, which contains the protein to be secreted from the transfected cells into the culture medium. Therefore, the level of SEAP activity is a direct quantification of protein-protein interactions, because its activity is directly proportional to the intracellular amount of mRNA and proteins [38,39]. The results showed that the CPB1-E interaction was relatively strong (Figure 1A) compared to the positive (Figure 1F) and negative controls (Figure 1B–E). The positive controls were Vero cells transfected with the pM3-VP16 plasmid, which encodes a Gal4 DNA binding domain fused to the transcription activator, VP16. On the other hand, negative controls consisted of mock-transfected controls (Figure 1B), empty pM and pVP16 controls (Figure 1C), pM with insert control (Figure 1D) and pVP16 with insert control (Figure 1E). These negative-control samples showed a minimal chemiluminescence level, which denoted the genuine interaction of CPB1-E.

### 3.3. CPB1 Co-Localizes with Dengue Virus 2 E Protein in the Endoplasmic Reticulum (ER) of Ae. aegypti Primary Midgut Cells

**Figure 2:**
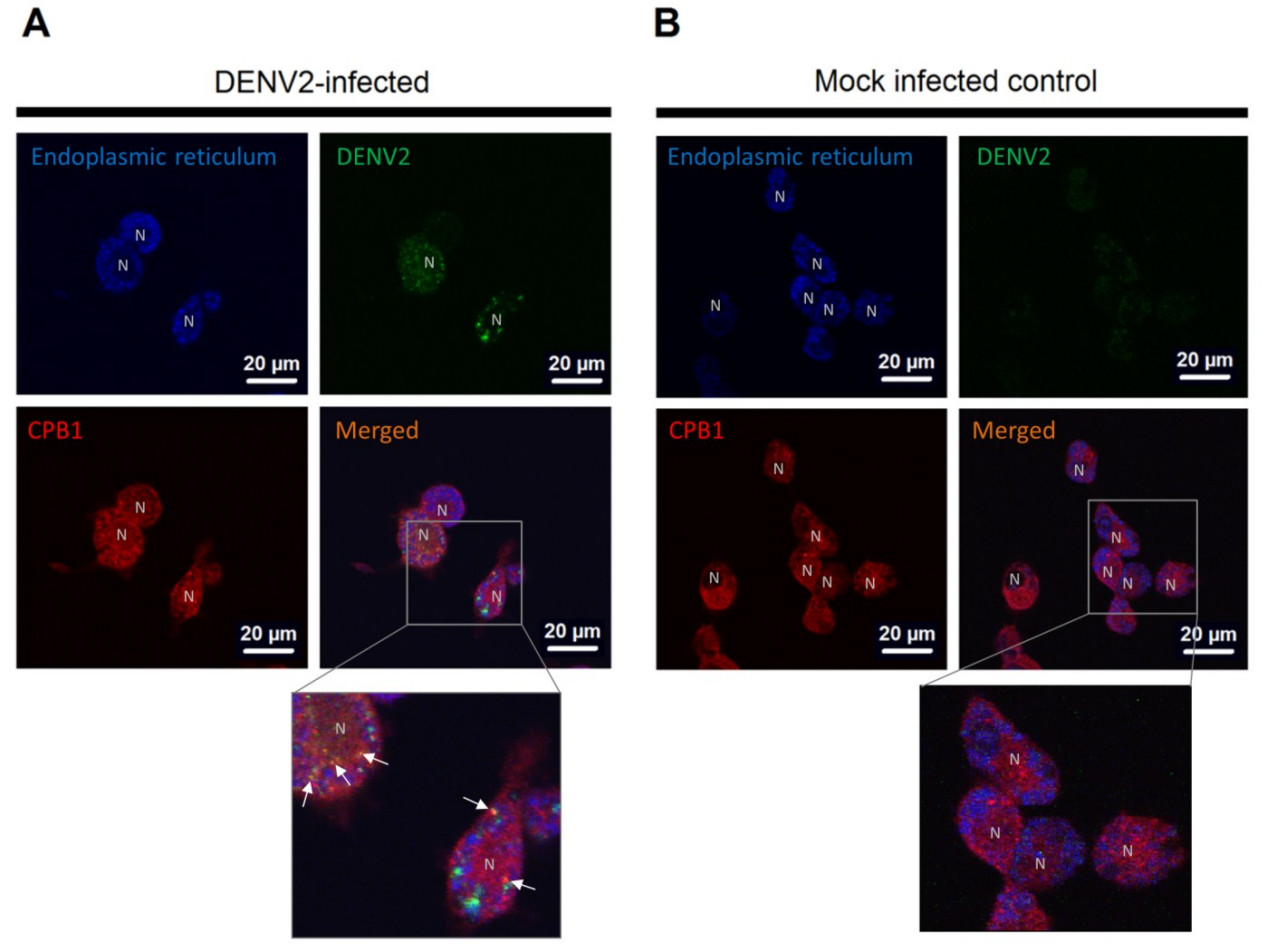
Cellular localization of CPB1 and DENV2 in the ER of *Aedes aegypti* primary midgut cells. (**A**) DENV2-infected mosquito primary midgut cells. (**B**) Mock-infected control. The midgut cells were isolated from female adult *Ae. aegypti* and maintained in MEM media supplemented with 10% FBS. At 48 hpi, the cells were stained with ER‑Tracker™ (blue, endoplasmic reticulum), fixed and stained for DENV2 (green, DENV2) and CPB1 (red, CPB1). The cells were mounted and viewed under a confocal microscope at 100× magnification with immersion oil (Olympus IX81). The ER, DENV2 and CPB1 images are overlaid into a merged image (merged). CPB1 is mainly observed throughout the cell cytoplasm (red), whereas DENV2 was found in DENV-infected cells (green). N, nuclei that were not stained to avoid confusion with the ER. White arrows in the close-up view indicate the co-localization of CPB1 and DENV2 within the blue ER regions. Bars = 20 µm.

The distribution of CPB1 in mosquito cells has not been previously assessed. Therefore, we investigated the cellular localization of CPB1 in *Ae. aegypti* midgut cells during DENV2 infection. Figure 2 shows the DENV2-infected (Figure 2A) and mock-infected primary midgut cells (Figure 2B). CPB1 was found throughout the cells, including the nuclei, regardless of the presence of DENV2 (Figure 2A,B, *CPB1*). Not all midgut cell types were susceptible to DENV2 infection, and the distribution of DENV2 varies between cell types (Figure 2A, *DENV2*). The CPB1 and E proteins co-localize in proximity to the nucleus, in the ER of DENV2-infected *Ae. aegypti* midgut cells (Figure 2A, white arrows in the close-up view). The ER in the cytoplasm was stained blue surrounding a “hollow” region in the majority of cells (Figure 2A,B, endoplasmic reticulum). These hollow regions are the nuclei, labelled “N”. No DENV2 was detected in the mock-infected control (Figure 2B, *DENV2*).

### 3.4. CPB1-E Protein Complexes Co-Immunoprecipitate from DENV2-Infected Ae. aegypti Primary Midgut Cells

**Figure 3:**
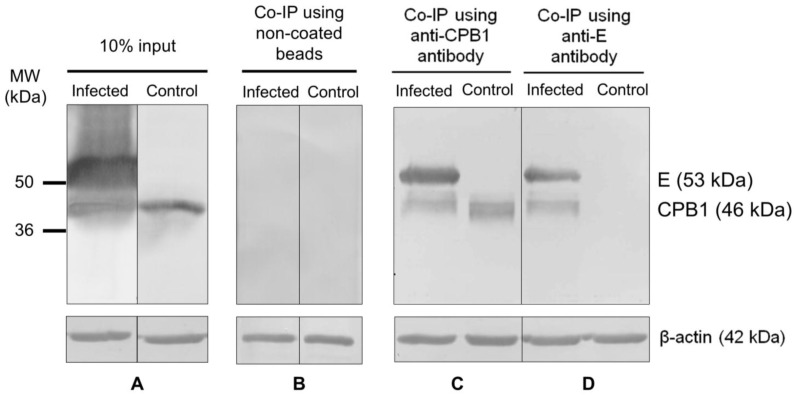
Co-immunoprecipitation of CPB1 and E proteins from DENV2-infected and mock-infected *Aedes aegypti* primary midgut cell lysate. SDS-PAGE and western blot were performed using Dynabeads^®^ conjugated with anti-CPB1 or anti-E antibodies. (**A**) 10% of the total cellular lysate. (**B**) Non-antibody-coated bead controls. Cell lysate derived from DENV2-infected *Ae. aegypti* primary midgut cells (infected). Control, cell lysate derived from mock-infected *Ae. aegypti* primary midgut cells. (**C**,**D**) Anti-CPB1 and anti-E antibodies pulled down the CPB1 and E protein from DENV2-infected samples (infected). For controls, anti-CPB1 antibody pulled down the CPB1 protein ((**C**), control), whereas no band was observed in the same sample when anti-E antibody was used ((**D**), control). Western blot analysis and β-actin protein levels of each sample are shown.

To further confirm the presence of CPB1-E protein complexes in *Ae. aegypti* during DENV2 infection, a co-immunoprecipitation assay was performed using the cell lysates of DENV2-infected *Ae. aegypti* primary midgut cells. In this assay, 10% input of total cellular lysate indicates the amount of CPB1 and E proteins in the lysate (Figure 3A). The non-coated beads served as the “resin-only” control, which showed its ultra-low background binding ability (Figure 3B). Anti-CPB1 antibody-coupled resin pulled down the 46-kDa CPB1 and the 56-kDa E protein of DENV2 (Figure 3C, infected). The same two bands were observed when anti-E antibody-coupled resin was used in an independent co‑immunoprecipitation experiment (Figure 3D, infected). In the uninfected controls, only CPB1 was pulled down when the anti-CPB1 antibody-coupled resin was used (Figure 3C, control). By contrast, no visible bands were observed when the anti-E antibody-coupled resin was used to co‑immunoprecipitate E or CPB1 proteins from the uninfected cells (Figure 3D, control). The multiple bands, which were consistently observed with CPB1, were degraded products of this protein. The β‑actin bands indicate that equal amounts of total cellular proteins were applied to each lane.

### 3.5. In Silico Docking Suggests Possible CPB1-E Interaction

**Figure 4:**
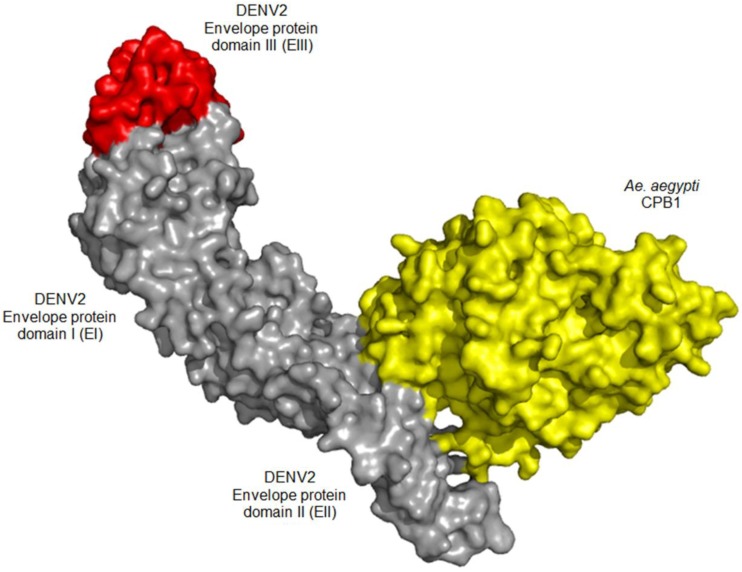
*In silico* molecular docking surface structure of the *Aedes aegypti* CPB1 and DENV2 E protein (PDB: 1OAN). The CPB1 protein structure was predicted using Iterative Threading ASSEmbly Refinement (I-TASSER). The CPB1-E protein rigid docking model was predicted using the PatchDock algorithm and further refined using the FireDock algorithm. The CPB1-E docking algorithm suggests the lowest binding energy of −22.7 kcal/mol. It also supports an interaction between CPB1 and the domain II of the E protein (EII), at residues Thr66, Asn67, Thr68, Lys122 and Val251. CPB1 is shown in yellow, and E in grey and red, where grey represents domains I and II and red represents domain III.

The docking algorithm supports CPB1-E interaction with a binding energy of −22.7 kcal/mol. The result also suggests that CPB1 binds to domain II of the E protein (EII), where CPB1 binds to Thr66, Asn67, Thr68, Lys122 and Val251. Figure 4 shows the predicted CPB1-E protein complex, with CPB1 illustrated in yellow, E in grey and red (grey: domain I and II; red: domain III) (Figure 4). 

### 3.6. Overexpression of CPB1 in Mosquito C6/36 Cells Results in Intracellular Accumulation of DENV2 Genomic RNA and Viral Components

**Figure 5:**
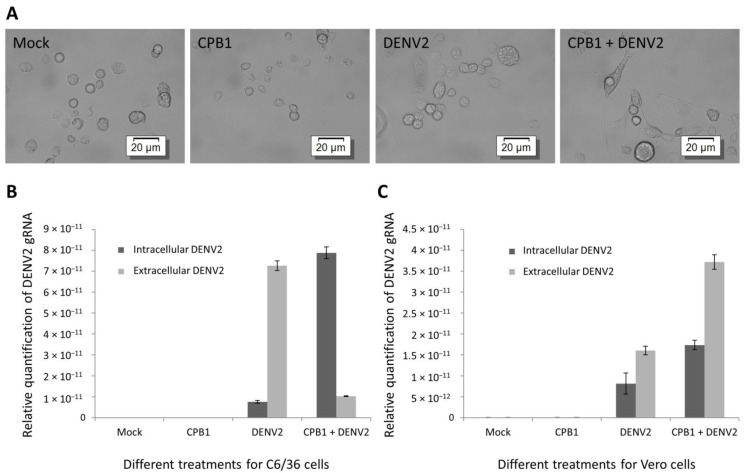
Intra- and extra-cellular DENV2 genomic RNA quantification of C6/36 and Vero cells during the upregulation of CPB1 expression. The cells were seeded in 24-well plates until 80% confluency, before pIB-CPB1 (for C6/36) or pcDNA-CPB1 (for Vero) was transfected. Protein expression was allowed for 48 hours, followed by DENV2 infection. Mock, mock-infected cells. CPB1, cells with upregulated CPB1 expression without DENV2 infection. DENV2, cells with DENV2 infection without CPB1 upregulation. CPB1 + DENV2, cells with DENV2 infection after CPB1 upregulation. (**A**) The cell morphology of C6/36 cells with different treatments. (**B**,**C**) The columns show the mean values and the error bars are the standard deviation (SD) of three samples (*n* = 3). Cellular lysates and media were harvested for RNA extractions at 48 hpi. DENV2 genomic RNA was quantified using relative RT-qPCR. Excessive CPB1 leads to the intracellular accumulation of DENV2 genomic RNA in C6/36 cells (*p* < 0.0001), with a significant decrease in virus released into the extracellular media (*p* < 0.0001). By contrast, Vero cells exhibited an opposite trend, in which despite the accumulation of DENV2 genomic RNA (*p* < 0.05), the amount of virus released was directly proportional to the intracellular genomic RNA accumulation (*p* < 0.05).

The morphology of C6/36 cells was altered, with increased size and cell elongation after DENV2 infection (Figure 5A). We also performed the relative quantification of intra- and extra-cellular DENV2 mRNA genomes of various C6/36 and Vero treatment groups by qPCR (Figure 5B,C). In both cell lines, DENV2 was not detected in either the cellular lysate or control sample media of non‑infected controls (Figure 5B,C, mock and *CPB1*). For DENV2-infected samples, a higher amount of DENV2 genomic RNA was detected in the media (extracellular) compared to the cellular lysate (intracellular) (Figure 5B,C, DENV2). However, after *Ae. aegypti* CPB1 upregulation, opposite outcomes were observed between C6/36 and Vero cell lines. For C6/36 cells, the DENV2 genomic RNA was primarily detected intracellularly and was 9.4-fold higher than the control (*p* < 0.0001), whereas significantly less DENV2 was released (*p* < 0.0001) (Figure 5B, CPB1 + DENV2). For Vero cells, overexpressed CPB1 resulted in an increase in intracellular and extracellular viral accumulation compared to Vero cells without CPB1 overexpression (*p* < 0.05) (Figure 5C, CPB1 + DENV2). Distinct outcomes were observed for DENV2‑infected C6/36 and Vero cells after CPB1 overexpression (Figure 5B,C, CPB1 + DENV2). The results implicate that CPB1 may exclusively regulate DENV2 replication in mosquito cells, in order to confer infectivity to *Ae. Aegypti* while maintaining the healthiness of its vector mosquitoes.

## 4. Discussion

In this study, we identified and localized CPB1, an *Ae. aegypti* protein that interacts with the E protein of DENV2. CPB1 binds to the DENV2 E protein in yeast two-hybrid screenings and mammalian two‑hybrid assay, and the interaction was confirmed by co-immunoprecipitation and co‑localization assays.

Various characteristics and functions of CPB1 have been reported in mosquito. The rapid induction of carboxypeptidase genes in the midgut of mosquito [40] and the upregulation of carboxypeptidase B1 and B3 in *Ae. aegypti* midgut have been demonstrated at 24 hours post blood meal (PBM) [41]. CPB1 is a well-known hydrolytic enzyme, which is involved in C-terminal peptide cleavage after blood ingestion [41]. Polyclonal antibodies against this mosquito-derived CPB1 antigen have also been shown to block the sexual development of malarial protozoan parasites in the midgut of *Anopheles stephensi* [42]. Carboxypeptidase D, a counterpart of CPB1, is a receptor for duck hepatitis B virus [43,44,45]. In addition, a recent study reported the interaction between carboxypeptidase A (CPA) and the DENV capsid (C) protein in the salivary gland of *Ae. aegypti* [6]. Based on these diverse roles of carboxypeptidases, we explored the functional relationship of *Ae. Aegypti*-derived CPB1 in response to DENV2 infections in the midgut cells of *Ae. aegypti* mosquito.

To elucidate the mechanisms involved in the interaction between CPB1 and the E protein, it was necessary to determine where this interaction occurred. Macrophages are a primary target for dengue virus infection. Harris *et al.* showed that a vitellogenic-like carboxypeptidase (CPVL) was highly localized in the endoplasmic reticulum (ER) of human THP-1 monocytic cells and macrophages [46]. Specifically, in macrophages, CPVL is glycosylated and retained in the ER. The results of our study are consistent with this finding. We showed that using *Ae. aegypti* primary midgut cells, the CPB1 also localises in the ER and is not the surface of the cells (Figure 2).

Studies have shown that CPB1 is naturally upregulated in mosquito midgut cells [41,47]. However, the natural upregulation of CPB1 during DENV infections in C6/36 and Vero cells has not been previously reported. To mimic the effect of upregulation of CPB1 in these cells, they were transfected with protein-expression plasmid containing the CPB1 cDNA insert, followed by DENV infection. Interestingly, overexpression of CPB1 protein in DENV2-infected C6/36 cells caused intracellular accumulation of viral genomes and immature virus (Figure 5B, CPB1 + DENV2). However, overexpression of CPB1 protein in Vero cells during DENV2 infection resulted in a higher amount of intracellular accumulation and extracellular virus released compared to DENV2-infected cells without CPB1 overexpression (Figure 5C).

It is hypothesized that, during DENV2 replication in *Ae. aegypti* midgut cells, CPB1 binds to the E proteins that are deposited on the ER intraluminal membrane or to the E proteins on immature virions. The interaction may reduce the encapsulation process of the newly-formed RNA into immature virus, resulting in DENV genomic RNA accumulation. Meanwhile, CPB1-E interaction on newly-assembled immature virus, including viruses that are stacked in the dilated ER cisterna, may hamper further processing into mature viruses in the trans-Golgi network. The accumulation of intracellular genomic materials or viruses causes cellular enlargement (Figure 5A, CPB1 + DENV2) and can, therefore, be explained by the accumulation of excessive DENV2 genomic RNA, DENV2 viral proteins and host cell materials [48], in addition to the cytopathic changes that occur during infection [49]. A similar effect of DENV genomic RNA accumulation during viral inhibition was also observed when autophagy was inhibited by spautin-1 (specific and potent autophagy inhibitor 1) [50]. Autophagy is required for optimal DENV RNA replication, and cellular lipid metabolism is necessary for energy production [51]. In addition, a reduction in the amount of secreted infectious virions was also observed in a study where the kinase inhibitor, SFV785, was used to dislocate the DENV envelope protein, thus blocking virus assembly [52].

We cannot exclude the possibility that the interaction between CPB1 and E could lead to inhibition of viral morphogenesis, such as viral glycoprotein processing, as described previously [53,54]. However, our *in silico* molecular modelling not only supports CPB1-E protein interaction, but also suggests that the residues of E protein to which CPB1 binds are Thr66, Asn67, Thr68, Lys122 and Val251. These residues are found in EII, and none of these residues are critical for the fusion-loop, hinge or E-M protein “latch” functionalities [55]. Further experimental results derived from mutational or isothermal titration calorimetry (ITC) analyses may support this computational finding.

The differences in the outcomes of DENV infections between cell lines suggest that there are dissimilarities in viral propagation pathways between insect and mammalian host systems. Many studies also confirmed the differential inhibition of DENV infection in mosquito and mammalian cells by the dissimilarity of the DENV E protein, polysaccharide composition between virions derived from mosquito and mammalian cells [56,57], different antiviral susceptibility to drug inhibitors of DENV2 entry [58] and endocytic uptake in Vero and C6/36 cells [59]. In this study, the differences in the outcomes could be due to several possible reasons. Firstly, CPB1 gene/protein used in the upregulation study is derived from mosquito. Sequence alignment conducted in our lab between the mammalian CPB1 (GenBank Accession Number: AAP36803.1) and mosquito CPB1 (GenBank Accession Number: AY590494.1) showed that the amino acid sequence homology is approximately 50%, thus indicating a significant differences in the protein characteristics. Therefore, the Vero cells might react differently to an insect CPB1 protein. Secondly, as a digestive enzyme for blood digestion after blood meal, CPB1 could therefore be suggested that the genes encoding the protein might be easily regulated in mosquitoes compared to Vero cells, where CPB1 has not been reported to be involved in any functional activities during DENV infection.

In this study, we demonstrated an anti-virus role for *Ae. aegypti* CPB1. Anti-viral mechanisms in insects have been widely studied, with reports showing delicate balances between viruses and the immune systems of insect vectors. These mechanisms include RNA interference (RNAi), Toll pathways, Janus kinase (JAK)-signal transducer and activator of transcription (STAT) (JAK-STAT) pathways and autophagy [60,61,62,63,64,65,66]. These systems control or contain viral growth, fine tune the virus titer and confer infectivity to the insect vector without impairing the health of the insect. Our results demonstrate the existence of a novel virus-control mechanism by CPB1 in the ER of *Ae. aegypti* midgut cells during DENV2 infection.

During a blood meal from a DENV-infected human, viral infection of midgut cells occurs. It is hypothesized that CPB1 upregulation after a blood meal [41] functions to regulate the intracellular accumulation and minimal extracellular secretion of infectious DENV2. The viral infection causes cytopathic changes due to viral RNA and immature virus accumulation, and these cells most likely progress to lysis or death, followed by virus release. The released viruses are mostly immature viruses, CPB1-bound immature viruses and a low titer of mature infectious viruses. This may favor the health of the vector mosquito while keeping the virus titer at a low level to reduce infection that may cause devastating damage to uninfected midgut and adjacent cells. Consistent with previous studies on DENV and Chikungunya virus expression profiles in different mosquito organs [67,68,69], mosquito salivary glands contained a lower virus titer compared to the midgut cells. As shown in this study, although the small amount of virus released from the midgut cells is not likely to cause secondary infection in other cell types in the mosquito host, the virus titer is strategically reduced for a delicate balance between viruses and the immune systems of insect vectors [60,61,62,63,64,65,66]. Our results demonstrate a novel virus-control mechanism using CPB1 in the ER of *Ae. aegypti* midgut cells during DENV2 infection.
